# Daylight saving time transitions and hospital treatments due to accidents or manic episodes

**DOI:** 10.1186/1471-2458-8-74

**Published:** 2008-02-26

**Authors:** Tuuli A Lahti, Jari Haukka, Jouko Lönnqvist, Timo Partonen

**Affiliations:** 1 Department of Mental Health and Alcohol Research, National Public Health Institute, Helsinki, Finland; 2 Department of Psychiatry, University of Helsinki, Helsinki, Finland

## Abstract

**Background:**

Daylight saving time affects millions of people annually but its impacts are still widely unknown. Sleep deprivation and the change of circadian rhythm can trigger mental illness and cause higher accident rates. Transitions into and out of daylight saving time changes the circadian rhythm and may cause sleep deprivation. Thus it seems plausible that the prevalence of accidents and/or manic episodes may be higher after transition into and out of daylight saving time. The aim of this study was to explore the effects of transitions into and out of daylight saving time on the incidence of accidents and manic episodes in the Finnish population during the years of 1987 to 2003.

**Methods:**

The nationwide data were derived from the Finnish Hospital Discharge Register. From the register we obtained the information about the hospital-treated accidents and manic episodes during two weeks before and two weeks after the transitions in 1987–2003.

**Results:**

The results were negative, as the transitions into or out of daylight saving time had no significant effect on the incidence of accidents or manic episodes.

**Conclusion:**

One-hour transitions do not increase the incidence of manic episodes or accidents which require hospital treatment.

## Background

Daylight saving time (DST) is used to balance the seasonal caused daylight exposure changes to the activity peaks of a population. It is important to explore the effects of transitions into DST to the public health as DST affects millions of people annually and its impacts are still widely unknown. Turning clock forwards (on spring) or backwards (on fall) by one hour presumably impacts our circadian rhythms. DST study can provide interesting knowledge about the importance of individual differences in the adjustment of changes in circadian rhythms. DST studies can also provide a useful indication of which type of people will generally find it easy to adjust [1] and which kind of people are at risen risk for developing symptoms caused by circadian rhythm disturbance.

Circadian clocks regulate the endogenous rhythms of vital functions. Circadian rhythms are not able to adjust instantaneously to sudden changes in sleep-wake cycle and thus sudden changes as transitions into DST can cause circadian rhythm disruptions [2]. Disturbance of sleep is the most sensitive sign about abnormalities in circadian function. It seems that in modern society most of us are chronically sleep-deprived [3,4] and due to this even small additional losses of sleep as caused by fall and spring transitions may have consequences for public and individual safety [5,6]. Swinging of the sleep-wake cycle may cause deficit of attention, memory and mood and lead to higher accident rates [3]. The consequences of sleepiness do have a major influence to the public health and thus should be taken very seriously. There are also researches that have shown DST reducing the amount of pedestrian and motor vehicle fatalities [7,8]. The results from different studies being contradictory, more studies of the phenomena are needed to understand accurately the effects of circadian rhythm disruption caused by fall and spring transitions into DST. It would also be very interesting to solve if there is some kind of a population to population variation in the capability of adjusting to seasonal light changes.

Both the Parliament and Council of the European Union (EU) have stated that personal injuries are one of the central health problems in societies (establishment agreement 1999, article 129). According to the Ministry of Social Affairs and Health of Finland, accidents cause annually costs of approximately 3.3 – 5 billion euros (counted for accidents happened for fifteen year olds and elder). It is presumable that if functioning systems to prevent the accidents can be developed it decreases the amount of accidents. Even small reduction in amount of accidents can cause major profits; if the number of hospital treated accidents can be reduced even 5%, 10 billion euros will be saved from treatment and social expenditures inside EU area [9]. To prevent accidents we should gather updated knowledge of circumstances causing accidents and do risk analyses for populations.

On our earlier studies we have shown that fall and spring transitions into DST may have disruptive effects on the rest-activity cycle of healthy adults [10]. Transitions reduce the duration of sleep and the efficiency of the deprived sleep [11]. Transitions can cause several significant changes in sleep variables as weakening the quality of the sleep and enhancing night time restlessness [12]. As it is known that sleep deprivation can trigger the appearance of mental illness it seems plausible that the effects of DST transitions can be even more disrupting among clinical patients inclined for sensitivity to abnormalities in regulation of the circadian clockwork. Disturbances of the circadian timing system are implicated in the pathogenesis of numerous clinical syndromes, including sleep and affective syndromes. The influence of light and circadian rhythms for mental illnesses is under serious research nowadays. It seems that patients with certain mental disorders suffer from disability to adjust the seasonal light changes [13-15]. This disability could explain some clinical features of manic-depressive illness such as the cyclic and seasonal appearance of the symptoms [16]. Manic episodes endure from few days to several weeks and usually require hospital treatment. Disturbances of sleep and circadian rhythms are essential features of manic episodes. Circadian research can help to optimize medical diagnostics and therapy and is remarkable for finding the reasons and treatments for symptoms caused by circadian rhythm disruption [17].

We hypothesized that transitions into and out of daylight saving time may influence the incidence of hospital-treated accidents and manic episodes. This hypothesis was based on the earlier findings of sleep disruption being able to cause accidents and manic episodes. We assumed a peak in the number of hospital-treated manic episodes and accidents after DST transitions.

## Methods

The material was derived from the Finnish Hospital Discharge Register which is supported by the National Research and Development Centre for Welfare and Health. Permission for the study was authorized by the National Research and Development Centre for Welfare and Health (STAKES), Helsinki, Finland, which is the holder of the nationwide data for the National Hospital Discharge Register. For the analysis, all the data were anonymous. The Finnish Hospital Discharge Register covers all mental and general hospitals, as well as in-patient wards of local health centers, military wards, prison hospitals and private hospitals in Finland. From the register we obtained the information about the hospital-treated accidents and manic episodes during two weeks before and two weeks after the transitions in 1987–2003. The into-transitions took place on the last Sunday of March during the study period. Prior to 1996, the out-of-transitions took place on the last Sunday of September. Since 1996, Finland as a member of the European Union adopted the last Sunday of October as the out-of-transition date.

Accidents and manic episodes were classified according International Classification of Diseases (ICD). Before 1995 the diagnoses were coded according to ICD-9, and for 1996–2003 they were coded according to ICD-10 (V01-X59 for accidents, and F30 or F31.0 to F31.2 for manic episodes). All periods of hospital treatment due to accidents and manic episodes appearing under the two preceding or two following weeks of spring and fall transitions were mapped out and from this data we gathered information as follows: the start and end days of treatment, diagnosis, the year of birth, sex and personal identity number.

We constructed a contingency table of frequencies of accidents and manic episodes in respect to year (1987–2003), season (spring/fall), age (6–16, 16–45, 45–65, 65–120), sex (male/female), geographical location (East, West, North, South), and period (before or after transitions). This table was modeled using Poisson regression with the cell frequency as response variable and the table marginal as the explanatory variable [18]. The significance of variables was tested using maximum likelihood tests. The effect of period (before or after transitions) was added into model after all other explanatory variables, thereby its effect was corrected for in respect to the other factors. Spring and fall transitions were analyzed separately.

## Results

Transitions into or out of DST had no significant effect on the incidence of accidents or manic episodes in the Finnish population during the years of 1987 to 2003 (see Tables 1 and 2).

**Table 1:** Analyses of deviance table for the log-linear models of hospital treatments due to accidents, explanatory variables added sequentially.

*Spring*			
	Df	Deviance	P value
Geographical location	3	5034	< 0.001
Age	4	26834	< 0.001
Sex	1	1	0.335
Year	15	1219	< 0.001
Period (before/after)	1	2	0.174
*Fall*			
			
	Df	Deviance	P value
Geographical location	3	5672	< 0.001
Age	4	22758	< 0.001
Sex	1	164	< 0.001
Year	15	797	< 0.001
Period (before/after)	1	2	0.309

Abbreviation: Df = Degrees of freedom

**Table 2:** Analyses of deviance table for the log-linear models of hospital treatments due to manic episodes, explanatory variables added sequentially.

*Spring*			
	Df	Deviance	P-value
Geographical location	3	174.61	< 0.001
Age	4	1689.36	< 0.001
Sex	1	16.61	< 0.001
Year	15	152.08	< 0.001
Period (before/after)	1	0.50	0.480
*Fall*			
			
	Df	Deviance	P-value
Geographical location	3	5672	< 0.001
Age	4	22758	< 0.001
Sex	1	164	< 0.001
Year	15	797	< 0.001
Period (before/after)	1	1	0.150

Abbreviation: Df = Degrees of freedom

In addition, we analyzed whether there was a significant effect of the different timing of fall transitions on the incidence of accidents or manic episodes, but there was none (data not shown).

## Discussion

The reason why not seen any impact of fall and spring transitions to manic episodes and accidents can derive from following factors; Genetic contributions are important explainers of the appearance of manic episodes and it seems that in a Finnish population the prevalence of manic episodes is quite low [19] thus the impact of DST can not be seen at our statistics. It might also be that turning clocks one hour back- or forwards has such slight impact to biological clock that its impacts can be kept under control with medication among those ones who already are treated because of mental illness. It has been pointed out earlier that wider transitions, such as caused by time zone crossings, can possibly play a role in the exacerbation of severe mental disorders [20]. This supports the hypothesis that disruptions of the circadian clockwork may trigger manic episodes. According to our results, however, it now seems that a one-hour transition is not disturbing enough that it would increase the incidence of manic episodes. Manic episodes instead occurred more often in fall than in spring time. This finding was not significant but still apparent. The fall transition out of DST leads to exposures to light earlier in the morning, and it may thereby cause phase advance on top of phase advanced positions in bipolar patients.

Concerning accidents the spring transition into DST might increase the number of minor accidents, such as small pedestrian casualties and tin crashes. However, since such accidents are unlikely to lead to admission, they will not be recorded in statistics. Accidents took place slightly more often in spring than in fall (table 1). This finding was not significant but still apparent. The reason for the increased incidence of accidents after the spring transition is probably caused by the loss of sleep. In spring the clocks are turned one hour forwards and thus the sleeper lose one hour, whereas in fall the clocks are turned backwards and thus the sleeper gains one more hour. Sleep deprivation tends to compromise reaction times and alertness levels and is therefore a marked factor predisposing to accidents.

## Conclusion

This study indicated no effect of DST transitions on admissions due to manic episodes or accidents.

## Competing interests

The author(s) declare that they have no competing interests.

## Authors' contributions

TAL made contributions to the analysis and interpretation to the drafting and writing of the manuscript.

JH made contributions to statistical modeling and analysis and to the drafting of the manuscript.

JL participated in the planning of drafting of the manuscript.

TP participated in the planning of the study, in the analysis of data, and in the drafting of the manuscript.

All authors red and approved the manuscript.

## Pre-publication history

The pre-publication history for this paper can be accessed here:


